# A Comprehensive Analysis of *FGF*/*FGFR* Signaling Alteration in NSCLC: Implications in Prognosis and Microenvironment

**DOI:** 10.1111/1759-7714.70016

**Published:** 2025-02-25

**Authors:** Ziling Huang, Leyao Li, Xu Cai, Shen Wang, Yun Jia, Yuan Li

**Affiliations:** ^1^ Department of Pathology Fudan University Shanghai Cancer Center Shanghai China; ^2^ Department of Oncology Shanghai Medical College of Fudan University Shanghai China; ^3^ School of Computer Science Fudan University Shanghai China; ^4^ Butiange Co. Ltd Shanghai China; ^5^ Alpha X Biotech (Beijing) Co. Beijing China

**Keywords:** *FGF/FGFR* signaling, microenvironment, NSCLC, prognosis

## Abstract

Fibroblast Growth Factor (FGF) ligands and their receptor have been identified as the potent target in non–small cell lung cancer (NSCLC). However, the clinicopathological and microenvironmental characteristics of *FGF/FGFR* in NSCLC remain poorly elucidated. Here, we summarize 4656 NSCLCs and analyze clinicopathological features in 478 *FGF/FGFR* altered cases. AI analysis and multiplex immunofluorescence staining are used to reveal microenvironment features. First, around 10.27% NSCLC carry *FGF/FGFR* variant. Squamous cell carcinoma (41.95%) is much more than adenocarcinoma (8.32%). In 118 pathogenic variant (PV) cases, the most frequent variant is *FGF/FGFR* copy number increase (83.05%), the second is *FGFR* gene fusion (11.86%). Surprisingly, *CCND1* always co‐amplifies with *FGF19* (100.00%). Furthermore, *FGF* PV is an independent risk factor for poor outcomes (overall survival: HR = 3.781, disease‐free survival: HR = 3.340). And one‐third of *FGFR3‐TACC3* fusion cases show clear cytoplasm in histology. Either *CCND1/FGF19* co‐amplification or *KRAS* co‐mutation is closely related to cigarette exposure, and *KRAS* co‐mutation acts as an independent factor of poor prognosis. Finally, the *FGF/FGFR1/NOTCH1* within *RB1* variant group has a remarkably high ratio of inner‐tumor CD8+ T cell infiltration, non‐exhausted T cells, exhausted T^CD8+PD‐1+LAG3−^ cells, and T_RM_^CD8+CD69+CD103+^cells. Overall, this study provides a comprehensive analysis of *FGF/FGFR* alteration in NSCLC. The *FGF/FGFR* alteration mainly arises in squamous cell carcinoma. Both *FGF* PV and *KRAS* are the independent factors for poor prognosis. To our knowledge, this is the first report to describe an inflamed microenvironment recruited by *NOTCH1*/*RB1* co‐mutation, indicating potential benefit from immunotherapy.

## Graphical abstract

This study summarizes 4656 lung cancer cases examined by next‐generation sequencing (NGS) and demonstrates that both *FGF* PV and *KRAS* are the independent factors for poor prognosis. This is the first report to describe an inflamed microenvironment recruited by *NOTCH1*/*RB1* co‐mutation subtype, which implies the potential benefit from immunotherapy. All these findings provide a comprehensive analysis about *FGF/FGFR* alteration in large‐scale Asian cohort.

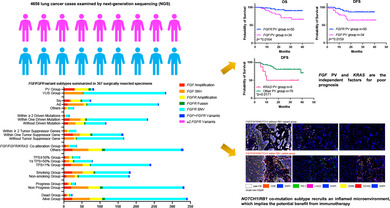

## 1 Introduction

Lung cancer is a major threatening issue for high mortality in worldwide [1, 2, 3]. Non–small cell lung cancer (NSCLC) is one of the common histological types in lung cancer, accounting for around 85% of the total cases [4, 5, 6]. The identification of the driver genes of NSCLC has therefore emerged as a prominent area of research. Except for the famous key drivers, like *EGFR*, *KRAS*, *HER2*, *BRAF*, *ALK*, *RET*, *ROS1*, and *c‐MET*, some potential targets reveal a new page in NSCLC anticancer therapy [5, 7, 8, 9, 10]. Revealing novel oncogenic driver variants is gradually becoming urgent in NSCLC.

Recently, fibroblast growth factor (FGF) ligands [11, 12, 13, 14, 15] and their receptors [13, 14, 15, 16, 17, 18] alteration have been identified as the potential and sensitive therapeutic targets for NSCLC. *FGFR* events, including amplification, fusion, and mutation, are the most prevalent type of *FGF/FGFR* alteration observed in lung cancer [14, 17]. There is a growing number of literatures that have reported patients who achieved disease response on FGFR inhibitor treatment [19, 20, 21, 22, 23, 24, 25], including the FDA‐approved pemigatinib, erdafitinib, infigratinib (BGJ398), and futibatinib (TAS‐120) specifically [18, 26, 27, 28]. However, there is a scarcity of studies on the survival outcomes and clinicopathological characteristics associated within the complex diversity of mutations in *FGF/FGFR* signaling pathway, and the roles of co‐mutations in modulating  the tumor microenvironment remain poorly understood.

Here, a total of 4656 NSCLC cases examined by next‐generation sequencing (NGS) are included in this study. We summarize 478 cases harboring *FGF/FGFR* alteration, and then analyze the relationship with clinicopathologic characteristics and prognosis. To our knowledge, this report is the first to characterize the tumor microenvironment associated with *FGF/FGFR* alterations, and offers comprehensive insights into the landscape of *FGF/FGFR* alterations in NSCLC.

## 2 Materials and Methods

### 2.1 Patients and Samples

A total of 4656 NSCLC tissue samples were examined by next‐generation sequencing (NGS), including 236 squamous cell carcinoma cases and 3978 adenocarcinoma cases. Among them, 478 harbor *FGF/FGFR* alteration, which consist of 367 surgically resected specimens and 111 biopsy specimens. The surgical samples were collected between April 1, 2020 and July 31, 2023 from Fudan University Shanghai Cancer Center, China. The clinical data and molecular results were obtained from the patients' electronic medical records. Patients who had a prior history of other malignancy or neoadjuvant chemotherapy were excluded. Clinicopathological parameters including age, gender, smoking history, histology, pathological stage, pathological subtype, and features such as visceral pleural invasion (VPI) and lymphovascular invasion (LVI) were recorded. Never‐smoker was defined as having smoked fewer than 100 cigarettes during the individual's lifetime. Histological type was classified according to the 5th edition of the World Health Organization (WHO) classification. The pathologic stage was determined by the 8th Edition of the American Joint Committee on Cancer (AJCC) staging system [29]. The study was approved by the Institutional Review Board of Fudan University Shanghai Cancer Center.

### 2.2 Multiplex Immunohistochemistry (mIHC) Staining

All the slides were deparaffinized in xylene for 10 min and repeated three times and rehydrated in absolute ethyl alcohol for 5 min and repeated twice, 95% ethyl alcohol for 5 min, 75% ethyl alcohol for 2 min, sequentially. Then the slides were washed with distilled water 3 times. A microwave‐oven was used for heat‐induced epitope retrieval; during epitope retrieval, the slides were immersed in boiling EDTA buffer (Alpha X Bio, Beijing, China) for 15 min. Antibody Diluent/Block (Alpha X Bio, Beijing, China) was used for blocking. The mIHC staining part was performed and analyzed according to a 6‐plex‐7‐color panel, and specifications (with primary antibodies used) are as the follows: Panel 1: CD4(ET1609‐52, HUA‐BIO, CHINA), FOXP3 (ab20034, Abcam, Cambridge, UK), TGF‐β (ab215715, Abcam, Cambridge, UK), FAP (ab207178, Abcam, Cambridge, UK), a‐SMA (ab124964, Abcam, Cambridge, UK) and PANCK (ZM0069, ZSGB‐BIO, CHINA). Panel 2: PD1(ZM0381, ZSGB‐BIO, CHINA), CD8 (ZA0508, ZSGB‐BIO, CHINA), CD69 (ab233396, Abcam, Cambridge, UK), CD103 (ET1611‐27, HUA‐BIO, CHINA), LAG3 (ab209236, Abcam, Cambridge, UK), and PANCK (ZM0069, ZSGB‐BIO, CHINA).

All the primary antibodies were incubated for 1 h at 37°C. Then slides were incubated with Alpha X Ploymer HRP Ms. + Rb (Alpha X Bio, Beijing, China) for 10 min at 37°C. Alpha X 7‐Color IHC Kit (Alpha X Bio, Beijing, China) was used for visualization. The correspondence between primary antibodies and fluorophores are listed: Panel 1: XTSA 480 (CD4), XTSA 520 (FOXP3), XTSA 570 (TGF‐β), XTSA 620 (FAP), XTSA 690 (a‐SMA) XTSA 780 (PANCK). Panel 2: XTSA 480 (CD8), XTSA 520 (PD1), XTSA 570 (CD69), XTSA 620 (CD103), XTSA 690 (LAG3) XTSA 780 (PANCK).

After each cycle of staining, heat‐induced epitope retrieval was performed to remove all the antibodies including primary antibodies and secondary antibodies. The slides were counter‐stained with DAPI for 5 min and enclosed in Antifade Mounting Medium (I0052; NobleRyder, Beijing, China). Axioscan7 (ZEISS, Germany) was used for imaging the visual capturing.

### 2.3 mIHC Image Analysis

Image analysis was performed with Halo (3.4, Indica Labs, United States). The random forest classifier was trained using manual annotation provided by the pathologist, including the classes ‘stroma’, ‘tumor,’ and ‘blank’. Cell phenotyping was performed using the analysis algorithm, Highplex FL, where the nuclear detection sensitivity and the minimum intensity threshold for positive cell detection based on staining localization (nuclear, cytoplasmic or membrane) were defined. And CD8+ cell localization was also identified by measuring the infiltration distances of CD8+ cell from tumor boundary using the infiltration analysis module in Halo.

### 2.4 HE Slides AI Analysis

The tumor microenvironment feature tertiary lymphoid structure (TLS) was recognized and evaluated by an optimized YOLOv8 AI model. The germinal center formation is defined as the feature of mature TLS. The number of both immature and mature TLS are all calculated and exported. The final number included for analysis is the average of all HE slides of tumor area of one case.

### 2.5 Molecular Analysis

Examination of *FGF/FGFR* signaling alteration by NGS used the Illumina NextSeq 500 platform with Oncoscreen Sequencing Panel (Burning Rock, Guangzhou, China), according to the standard protocol.

### 2.6 Statistic

Statistical analyses were performed using the software package Statistical Package for Social Sciences, version 20.0, for Windows (SPSS). Chi‐square or Fisher's exact tests were used to identify the influence of clinicopathological parameters on different *FGF/FGFR* signaling variants. Pearson's correlation coefficient was calculated for the *FGF/FGFR* alterations and the paired mutations or PD‐L1 expression of NSCLC. Overall survival (OS) and disease‐free survival (DFS) were estimated using the Kaplan–Meier method/Log‐rank (Mantel–Cox) test, and prognostic factors were assessed using Cox proportional hazards survival regression analysis. OS: Length of time between the surgery date to the death date (including any cause) or the last follow‐up date. DFS: Length of time from the date of surgery to the timepoint of first recurrence/metastasis, death from any cause, or last follow‐up. All statistical values were determined using two‐tailed statistical analyses, and a *p* value < 0.05 was considered statistically significant.

## 3 Results

### 3.1 NGS Analyses Reveal Diversity in *FGF/FGFR* Alterations

**FIGURE 1:**
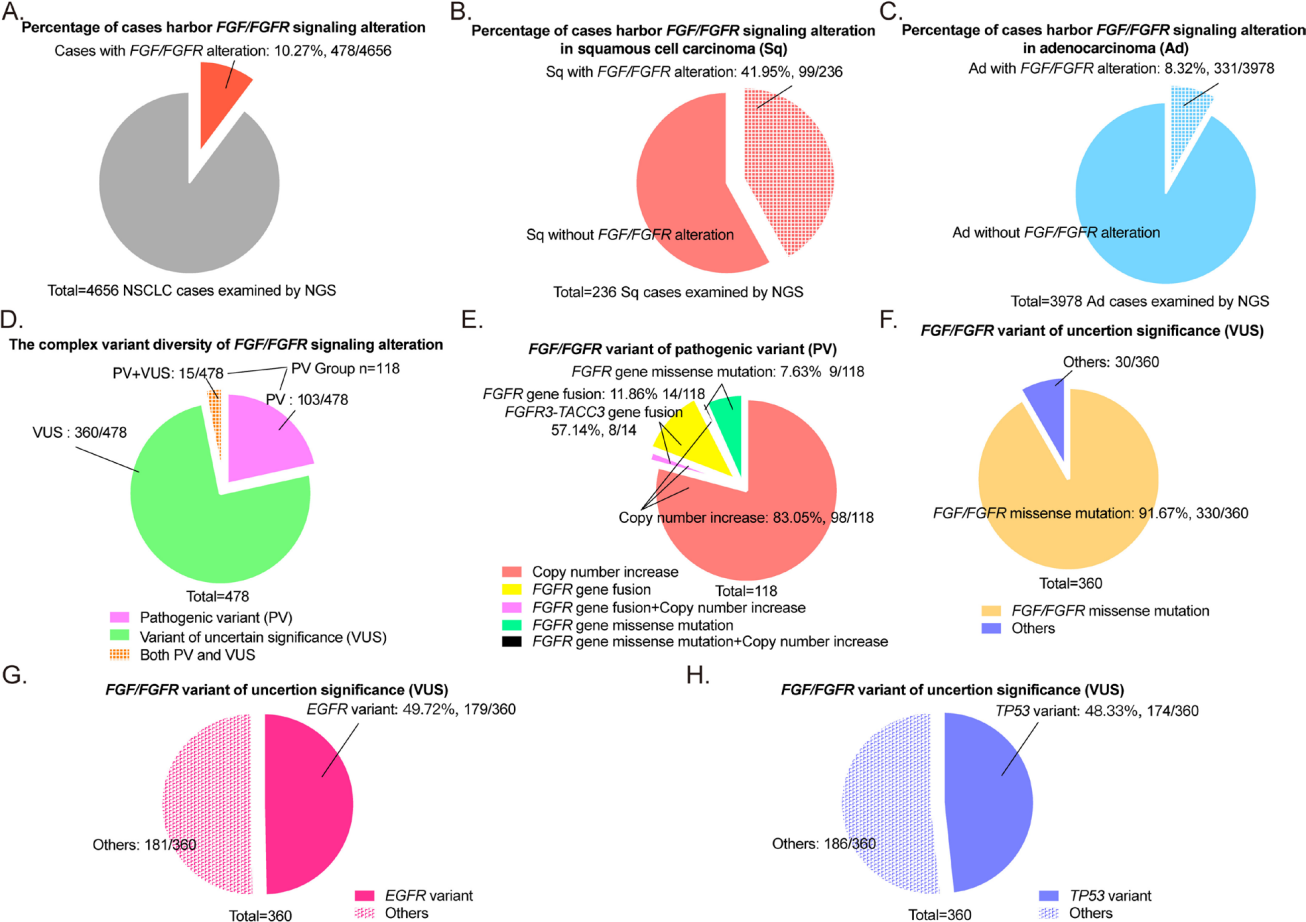
*FGF/FGFR* signaling alteration shows diversity by NGS examination. (A) The *FGF/FGFR* variation rate is around 10.27% (478/4656) in NSCLC examined by next‐generation sequencing (NGS). (B) The percentage of *FGF/FGFR* alteration in squamous cell carcinoma (Sq) is 41.95% (99/236). (C) The percentage of *FGF/FGFR* alteration in adenocarcinoma (Ad) is 8.32% (331/3978). (D) In the total 478 *FGF/FGFR* alteration cases, 24.69% (118/478) cases harbor pathogenic variant (PV), fifteen of them also harbor variant of only uncertain significance (VUS, 3.14%,15/478). The other 75.31% cases (360/478) carry VUS. (E) In the 118 PV cases, *FGF/FGFR* copy number increase accounts for 83.05% (98/118, red+magenta+black), *FGFR* gene fusion ratio is 11.86% (14/118, yellow+magenta), more than half cases carry *FGFR3‐TACC3* gene fusion (57.14%, 8/14). And the percentage of *FGFR* gene missense mutation is around 7.63% (9/118, green+black). Inside these cases, two of them carry both *FGFR* gene fusion and copy number increase (magenta). One harbor both *FGFR* gene missense mutation and copy number increase (black). (F) In the 375 cases within VUS, missense mutation of *FGF/FGFR* genes accounts for 91.67% (330/360). (G) The ratio of VUS cases co‐mutated with *EGFR* is around 49.72% (179/360). (H) The ratio of VUS cases co‐mutated with *TP53* variant is about 48.33% (174/360).

First, in 4656 cases, 478 harbor *FGF/FGFR* alteration. The total variation rate is around 10.27% (478/4656) in NSCLC examined by NGS (Figure 1A). Most *FGF/FGFR* variants are found in squamous cell carcinoma (Sq, 41.95%, 99/236, Figure 1B) rather than adenocarcinoma (Ad, 8.32%, 331/3978, Figure 1C). Furthermore, around 24.69% (118/478) cases harbor pathogenic variant (PV), 15 of them also harbor variant of only uncertain significance (VUS, 3.14%,15/478). The other 75.31% cases (360/478) carry VUS (Figure 1D).

In the 118 PV cases, *FGF/FGFR* copy number increase is the most frequent one (83.05%, 98/118), including *FGF19*, *FGF4*, *FGF3*, *FGFR1*, and *FGFR2* genes (Figure 1E). The second one is *FGFR* gene fusion, accounting for 11.86% (14/118) of 118 cases. *FGFR3‐TACC3* fusions is detected in more than half of the tissue samples (57.14%, 8/14, Figure 1E). And the third one is *FGFR* gene missense mutation (7.63%, 9/118, Figure 1E). Furthermore, the most common co‐mutation genes are *TP53*, *CCND1*, and *CDKN2*. Interestingly, the findings reveal that *CCND1* amplifications are invariably accompanied by amplifications in the *FGF19*  gene (100.00%, 43/43).

Moreover, among the 375 cases within VUS, the majority is missense mutation of *FGF/FGFR* genes (91.67%, 330/360, Figure 1F). Cases harbored VUS are most co‐mutated with *EGFR* (49.72%, 179/360, Figure 1G) and *TP53* variant (48.33%, 174/360, Figure 1H).

To sum up, the *FGF/FGFR* alteration mainly arises in squamous cell carcinoma. *FGF/FGFR* copy number increase has priority over other variants in PV samples, while missense mutation is the most common one in VUS cases. Besides, *FGF19* always co‐amplify with *CCND1* gene in PV cases.

### 3.2 The Survival and Clinicopathological Characteristics in Surgically Resected Specimens Within *FGF/FGFR* Variant

Second, to better understand the survival and clinicopathological characteristics in *FGF/FGFR* variant, we analyze 367 surgically resected specimens, including 84 PV and 283 VUS cases. Most PV cases are Sq, while the majority of VUS are Ad (Sq: 61.90%, 52/84, Ad: 85.87%, 243/283, *p* < 0.05, Table 1). The majority of cases haboring PV are current smokers or ever‐smokers (PV: 78.57%, 66/84, VUS: 42.05%, 119/283, *p* < 0.05, Table 1).

**TABLE 1:** The clinicopathological characteristics in 367 surgically resected specimens within *FGF/FGFR* variant.

Characteristics	Total (*n* = 367)	PV (*n* = 84)	VUS (*n* = 283)	*p*
Age				0.135^a^
Median (IQR)	63.0 (55.0–68.0)			
≤ 63.0	194 (52.86%)	38 (45.24%)	156 (55.12%)	
> 63.0	173 (47.14%)	46 (54.76%)	127 (44.88%)	
Sex				< 0.001^a^
Female	146 (39.78%)	13 (15.48%)	133 (47.00%)	
Male	221 (60.22%)	71 (84.52%)	150 (53.00%)	
Tumor size				< 0.001^a^
< 1.3 (25th percentile)	83 (22.62%)	3 (3.57%)	80 (28.27%)	
≥ 1.3 and < 2.0 (50th percentile)	79 (21.53%)	8 (9.52%)	71 (25.09%)	
≥ 2.0 and < 3.5 (75th percentile)	106 (28.88)	23 (27.38%)	83 (29.33%)	
≥ 3.5	99 (26.98%)	50 (59.52%)	49 (17.31%)	
Histology				< 0.001^a^
Adenocarcinoma	263 (71.66%)	20 (23.81%)	243 (85.87%)	
Squamous carcinoma	76 (20.71%)	52 (61.90%)	24 (8.48%)	
Other	28 (7.63%)	12 (14.29%)	16 (5.65%)	
Differentiation				< 0.001^a^
Well differentiated	54 (14.71%)	2 (2.38%)	52 (18.37%)	
Moderately differentiated	124 (33.79%)	17 (20.24%)	107 (37.81%)	
Poorly differentiated	189 (51.50%)	65 (77.38%)	124 (43.82%)	
pStage				< 0.001^a^
I	265 (72.21%)	44 (52.38%)	221 (78.09%)	
II	36 (9.81%)	13 (15.48%)	23 (8.13%)	
III	55 (14.99%)	23 (27.38%)	32 (11.31%)	
IV	11 (3.00%)	4 (4.76%)	7 (2.47%)	
TNM_T stage				< 0.001^a^
T1	252 (68.66%)	30 (35.71%)	222 (78.45%)	
T2	80 (21.80%)	34 (40.48%)	46 (16.25%)	
T3	22 (5.99%)	13 (15.48%)	9 (3.18%)	
T4	13 (3.54%)	7 (8.33%)	6 (2.12%)	
TNM_N stage				0.093^a^
N0	304 (82.83%)	63 (75.00%)	241 (85.16%)	
N1	23 (6.27%)	8 (9.52%)	15 (5.30%)	
N2	40 (10.90%)	13 (15.48%)	27 (9.54%)	
Venous/lymphatic invasion				0.007^a^
Absent	270 (73.57%)	52 (61.90%)	218 (77.03%)	
Present	97 (26.43%)	32 (38.10%)	65 (22.97%)	
Nerve invasion				0.012^b^
Absent	346 (94.28%)	74 (88.10%)	272 (96.11%)	
Present	21 (5.72%)	10 (11.90%)	11 (3.89%)	
Pleural invasion				0.066^a^
Absent	330 (89.92%)	71 (84.52%)	259 (91.52%)	
Present	37 (10.08%)	13 (15.48%)	24 (8.48%)	
STAS				0.017^a^
Absent	283 (77.11%)	73 (86.90%)	210 (74.20%)	
Present	84 (22.89%)	11 (13.10%)	73 (25.80%)	
Smoking history				< 0.001^a^
Absent	182 (49.59%)	18 (21.43%)	164 (57.95%)	
Present	185 (50.41%)	66 (78.57%)	119 (42.05%)	
PD‐L1 TPS				0.061^a^
TPS < 1%	242 (65.94%)	48 (57.14%)	194 (68.55%)	
1% ≤ TPS < 50%	80 (21.80%)	20 (23.81%)	60 (21.20%)	
TPS ≥ 50%	45 (12.26%)	16 (19.05%)	29 (10.25%)	

*Note:* Data are presented as *n* (%).

Abbreviations: IQR, interquartile range; PD‐L1, programmed cell death protein‐1 ligand; PV, pathogenic variant; STAS, spread through air spaces; TNM, tumor‐node‐metastasis; TPS, tumor proportion scoring; VUS, variant of only uncertain significance.

^a^ Pearson chi‐square is used for calculation.

^b^ Continuity correction is used for calculation.

Besides, the patients harboring PV tends to present with later pStage (II‐IV stage), whereas those carrying VUS mostly present with pStage I tumors (*p* < 0.05, Table 1). Additionaly, the tumors harboring PV tend to exhibit venous/lymphatic invasion (PV: 38.10%, 32/84, VUS: 22.97%, 65/283, *p* < 0.05, Table 1) as well as nerve invasion (PV: 11.90%, 10/84, VUS: 3.89%, 11/283, *p* < 0.05, Table 1). Nevertheless, the occurrence of spread through air spaces (STAS) is slightly more common in the VUS group than in the PV group (PV: 13.10%, 11/84, VUS: 25.80%, 73/283, *p* < 0.05, Table 1).

As described, *FGF/FGFR* PV is the primary variant in Sq, while *FGF/FGFR* VUS is the prevalent alteration in the Ad tissues. PV cases have a higher risk of smoking exposure, increased venous/lymphatic invasion, and later pStage. Altogether, these findings indicates that tumors harboring PV tends to be more aggressive in nature.

### 3.3 *FGF* Variant Is a Predictor of Poor Prognosis

**FIGURE 2:**
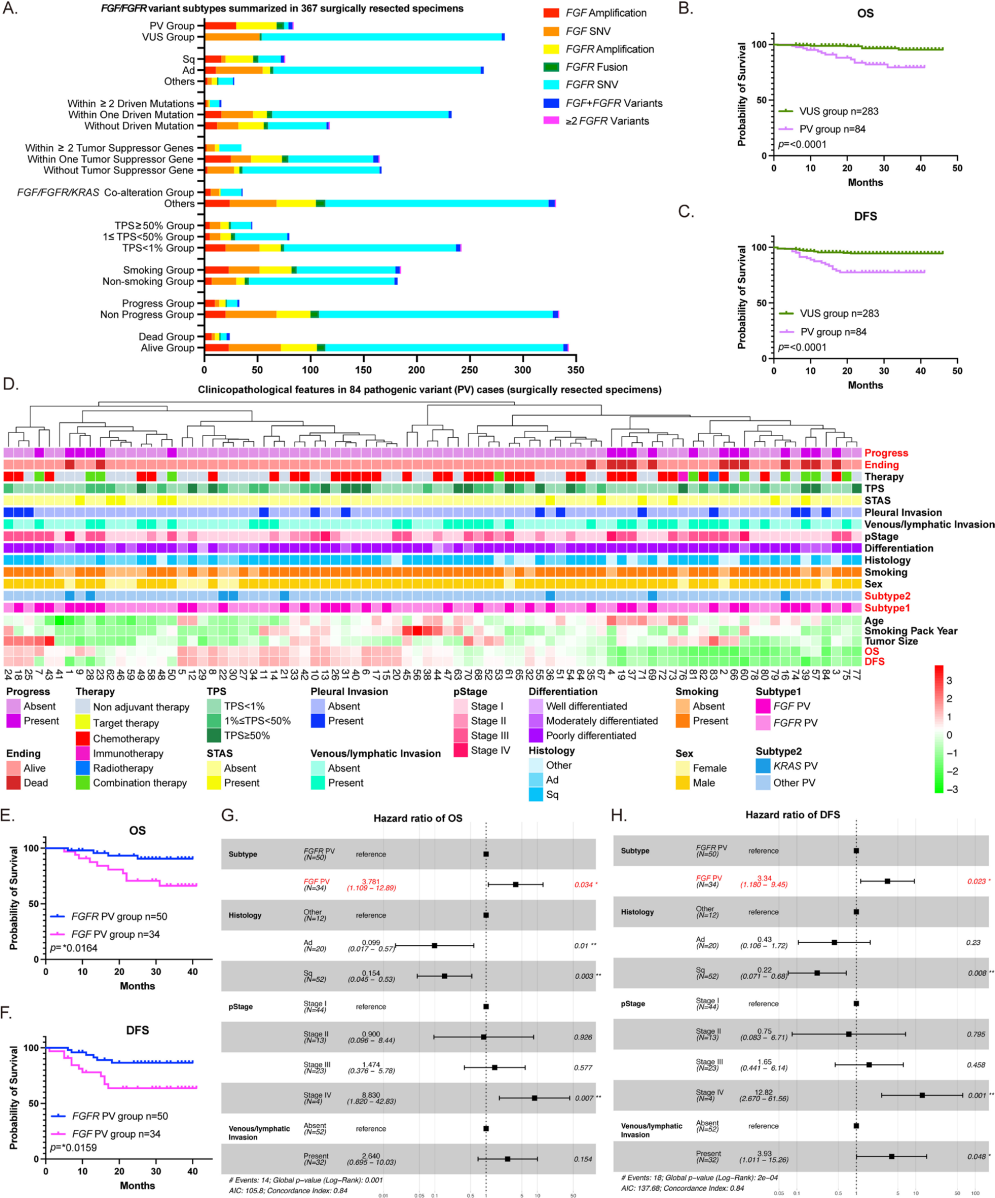
Survival curve shows the shorter OS and DFS in *FGF* PV group compared to *FGFR* PV ones. (A) The summary of *FGF/FGFR* variant subtypes in 367 surgically resected specimens. The variants include *FGF* amplification, *FGF* (single nucleotide variant) SNV, *FGFR* amplification, *FGFR* fusion, *FGFR* SNV, *FGF* + *FGFR* variants, ≥ 2 *FGFR* variants. (B) The overall survival (OS) is dramatically shorter in pathogenic variant (PV) group in contrast to VUS group. The survival rate is 79.47% in PV group while 95.24% in VUS group (****p < 0.0001). (C) The disease‐free survival (DFS) is dramatically shorter in PV group compared to VUS group. The survival rate is 77.47% in PV group while 94.55% in VUS group (****p < 0.0001). (D) The heatmap of clinicopathological features in 84 PV surgically resected specimens, including nineteen parameters. (E) The OS is dramatically shorter in *FGF* PV group in contrast to *FGFR* PV group. The survival rate is 66.21% in *FGF* PV group while 90.49% in *FGFR* PV group (*p < 0.05). (F) The DFS is dramatically shorter in *FGF* PV group compared to *FGFR* PV group. The survival rate is 63.75% in *FGF* PV group while 86.57% in *FGFR* PV group (*p < 0.05). (G) The multivariate analysis shows the hazard radio (HR) of OS in PV subtype, histology, pStage and venous/lymphatic invasion. The HR in *FGF* PV group is as high as 3.781 (95% CI: 1.109–12.890, *p < 0.05). (H) The multivariate analysis shows the HR of DFS in PV subtype, histology, pStage and venous/lymphatic invasion. The HR in *FGF* PV group is as high as 3.340 (95% CI: 1.180–9.450, *p < 0.05).

The effects of *FGF/FGFR* alterations on disease prognosis are elucidated by comprehensive analysis of the *FGF/FGFR* variant subtypes. The findings reveal that *FGFR* gene fusions are strongly associated with better prognosis, followed by *FGF* amplification and co‐mutaions in tumor suppressor genes and *FGF/FGFR* (Figure 2A and Table S1, *p* < 0.0001). A significantly shorter overall survival (OS) and DFS are shown in PV cases compared to VUS ones (OS and DFS: *p* < 0.0001, Figure 2B,C). Then we summarize in detail of the 84 *FGF/FGFR* PV cases, which are supposed associated with more aggressive tumor behavior (Figure 2D). Among these patients, 19 (22.62%) exhibit disease progression and 14 (16.67%) die during follow‐up (median follow‐up of 28 months). The specific factor which accounts for the poor prognosis is still unclear. To better illustrate this point, we compare between two subgroups, which are *FGF* variant (34/84, four cases carry both *FGF* and *FGFR* variants) and *FGFR* alteration (50/84). Though the clinicopathological parameter analysis indicates no big difference between these two groups (Table S2), the survival analysis uncovers a significant differences between the groups. Both OS and DFS are dramatically shorter in *FGF* PV group instead of *FGFR* PV group (OS: *p* = 0.0164 and DFS: *p* = 0.0159, Figure 2E,F). Univariate analysis of both OS and DFS reveal that the *FGF* PV is a statistically significant prognosis factor (OS: hazard radio (HR) =3.747, 95% CI: 1.173–11.970; DFS: HR = 3.431, 95% CI: 1.287–9.147, *p* < 0.05, Tables S3 and S4). The multivariate analysis further confirms that the presence of *FGF* PV is an independent prognostic risk factor for NSCLC (OS: HR = 3.781, 95% CI: 1.109–12.890; DFS: HR = 3.340, 95% CI: 1.180–9.450, *p* < 0.05, Figure 2G,H).

### 3.4 Distinct Histological Features associated with *FGFR* Alterations

**FIGURE 3:**
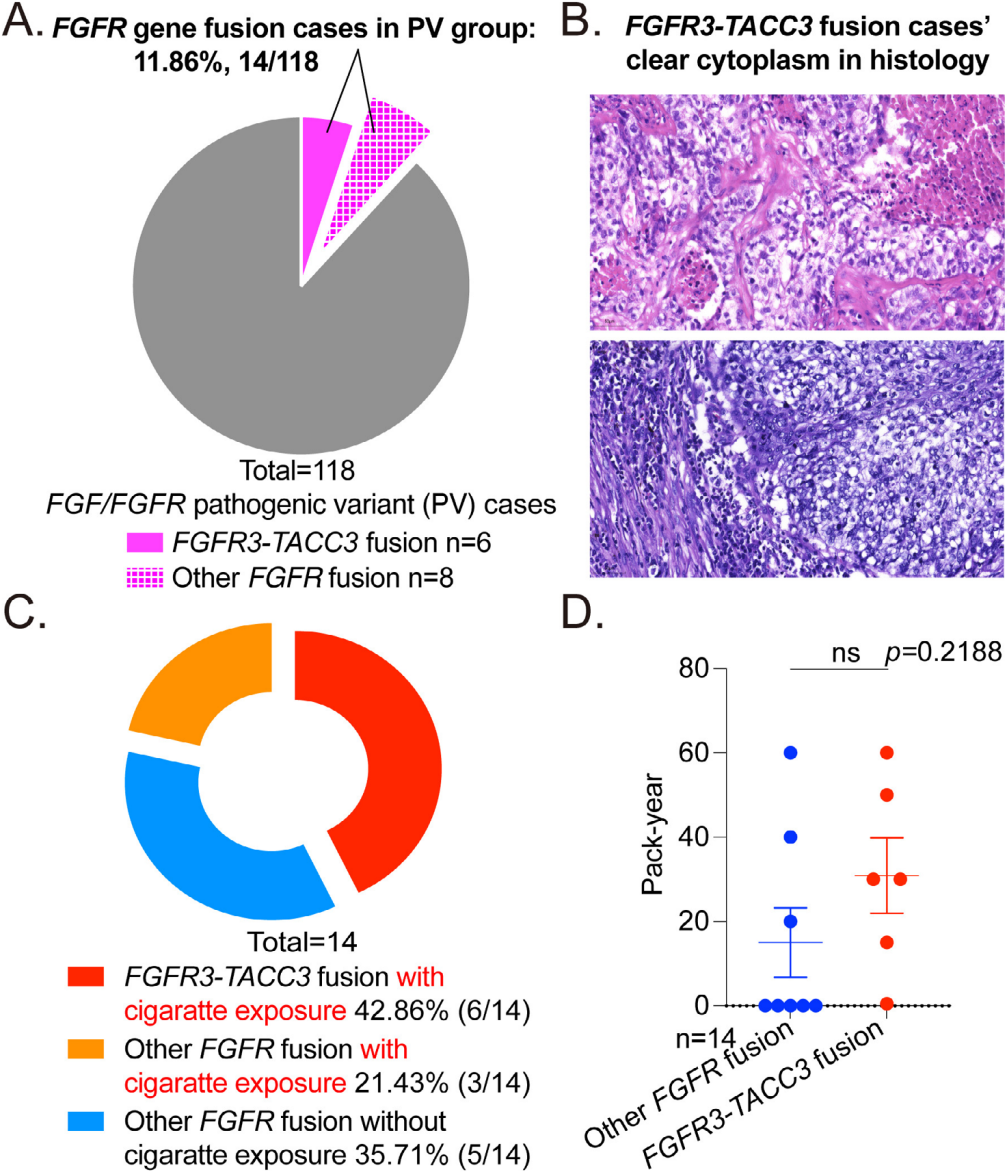
Survival curve shows the shorter OS and DFS in *FGF* PV group compared to *FGFR* PV ones. (A) In 118 PV cases, the proportion of *FGFR* gene fusion is about 11.86% (14/118). The most common *FGFR* gene fusion subtype is *FGFR3‐TACC3* (42.86%, 6/14). (B) The representative view of clear cytoplasm in *FGFR3‐TACC3* fusion cases (33.33%, 2/6, scale bar = 50 μM). (C) In fourteen *FGFR* gene fusion cases, 42.86% is *FGFR3‐TACC3* fusion with cigarette exposure, 21.43% is other *FGFR* fusion with cigarette exposure, the other 35.71% is other *FGFR* fusion without cigarette exposure. (D) There is no significant difference of cigarette pack‐year between *FGFR3‐TACC3* fusion and other *FGFR* fusion cases (*p* = 0.2188).

Although *FGFR* variant is not a predictor for poor prognosis in the group harboring PV, they remain a significant concern due to their potential as promising rug targets. An interesting thing is that *FGFR* gene fusion, ranked as the second alteration in 118 PV cases (11.86%, 14/118), exhibits a distinctive clinicopathological feature. Around 42.86% of *FGFR* gene fusion subtype is *FGFR3‐TACC3* (6/14, Figure 3A). And all these samples harboring the *FGFR3‐TACC3* subtype display poor differentiation (100%, 6/6). Interestingly, about one‐third tends to display clear cytoplasm in histology (33.33%, 2/6, Figure 3B). In contrast with the other fusion types, *FGFR3‐TACC3* fusion is tightly related to later TNM_T stage (T2‐T3: 100.00%, 6/6, *p* = 0.020, Table S5), and higher risk with cigarette exposure (*p* < 0.05, Figure 3C,D and Table S5).

### 3.5 Association of Co‐mutant Genes and FGF/FGFR Variants With Smoking Status and Poor Prognostic Risk

**FIGURE 4:**
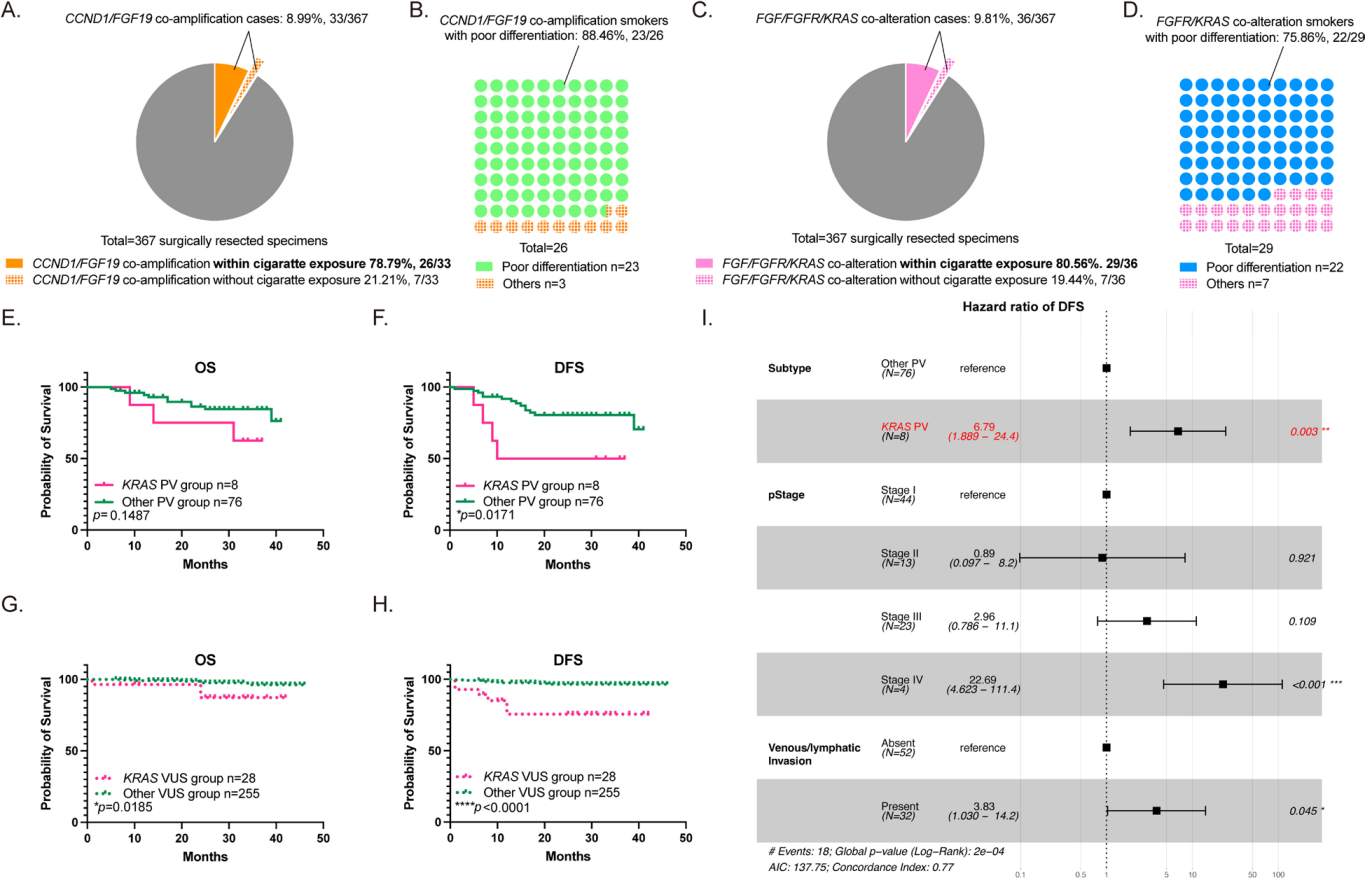
Co‐mutation genes reveal the smoking and poor prognosis risk involved in *FGF/FGFR* variant. (A) In 367 surgically resected specimens, *CCND1/FGF19* co‐amplification accounts for 8.99% (33/367). The priority of *CCND1/FGF19* co‐amplification PV cases is smoker or ever smoker (78.79%, 26/33). (B) Approximately, 88.46% cases exhibit poor differentiation (23/26). (C) Co‐mutant gene *KRAS* occupies around 9.81% in 367 surgically resected specimens (36/367). These cases are proved to have high ratio of cigarette exposure (80.56%, 29/36). (D) The 75.86% among these smokers have poor differentiation (22/29). (E) In survival analysis, shorter OS is observed in within *KRAS* co‐mutation PV group. The survival rate is 50.00% in *KRAS* PV group while 70.40% in other PV group (*p* = 0.1478). (F) Shorter DFS is observed in within *KRAS* co‐mutation PV group. (G) Shorter OS is observed in within *KRAS* co‐mutation group. The survival rate is 87.24% in *KRAS* VUS group while 96.19% in other VUS group (*p < 0.05). (H) Shorter DFS is observed in within *KRAS* co‐mutation group. The survival rate is 75.59% in *KRAS* VUS group while 96.49% in other VUS group (****p < 0.0001). (I) The multivariate analysis shows the HR of DFS in PV subtype, pStage and venous/lymphatic invasion. The HR in *KRAS* PV group is as high as 6.790 (95% CI: 1.889–24.400, **p < 0.01).

Furthermore, the co‐mutation genes also act as the risk factor involved in *FGF/FGFR* alteration. As we mentioned above, cigarette exposure plays an important role in *FGF/FGFR* variant samples (50.41%, 185/367), especially PV group (78.57%, 66/84, Table 1). Interestingly, among some paired variants, the smoking status is tightly linked to poor morphology features. For example, the majority of cases harboring PV and *CCND1/FGF19* co‐amplifications include smokers or ever smokers (78.79%, 26/33, Figure 4A), and the majority of tissues exhibit poor differentiation (88.46%, 23/26, Figure 4B). Besides, another co‐mutant gene *KRAS*, which occupies around 9.81% (36/367, Figure 4C) in surgically resected specimens, is proved to have high ratio of cigarette exposure (80.56%, 29/36, Figure 4C). Among these smokers, 75.86% patients are found within poor differentiation (22/29, Figure 4D). In survival analysis, shorter OS and DFS are observed in *KRAS* co‐mutation group (PV: Figure 4E,F, VUS: Figure 4G,H). Univariate analysis detects that *KRAS* PV is a predictor for poor DFS and not yet OS (DFS: HR = 3.279, 95% CI: 1.076–9.991, *p* < 0.05, Tables S3 and S4). And multivariate analysis proves that *KRAS* PV is an independent risk factor (DFS: HR = 6.790, 95% CI: 1.889–24.400, *p* < 0.05, Figure 4I).

In sum, neighboring gene mutation on 11p13 region (*CCND1/FGF19* co‐amplification) is a gene event tightly related to smoking and poor differentiation. *KRAS* co‐mutation is proved to be an independent risk factor of poor DFS in PV patients.

### 3.6 *FGF/FGFR* and *NOTCH1/RB1* Co‐Mutations Promote an Inflammatory Microenvironment, Contributing to a “Hot” Tumor Phenotype

**FIGURE 5:**
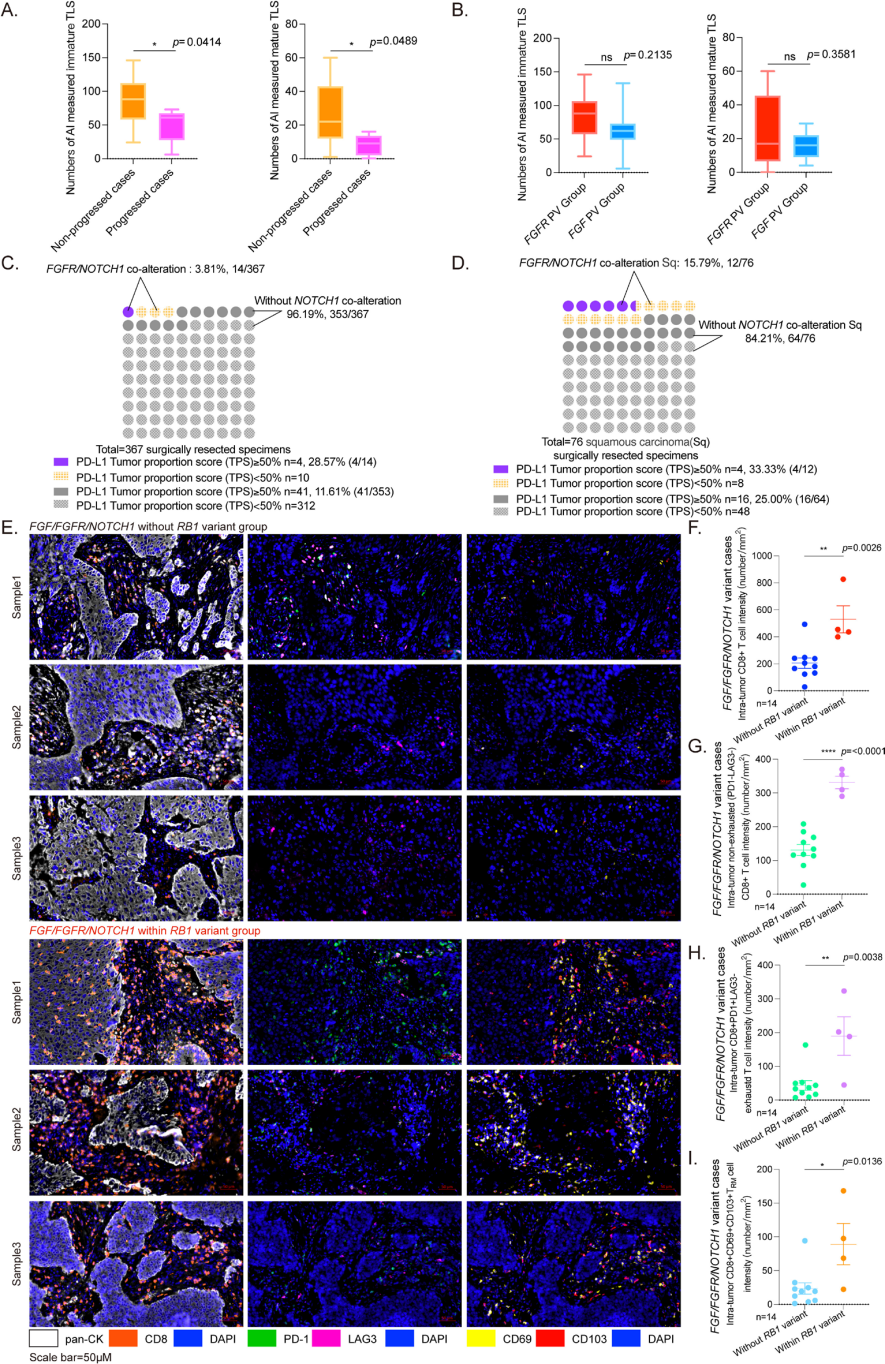
(A) *AI* ‐based model detects both high immature and mature TLS numbers in progressed case rather than non‐progressed ones in PV group (*p < 0.05). (B) There is no significant difference between *FGFR* and *FGF* variant cases for both immature (*p* = 0.2135) and mature TLS numbers (*p* = 0.3581). (C) *FGF/FGFR* co‐mutant within *NOTCH1* accounts for 3.81% (14/367) in 367 surgically resected specimens, including 8 PV and 6 VUS cases. Co‐mutant within *NOTCH1* samples has an obvious high PD‐L1 expression ratio compared to without *NOTCH1* cases (*NOTCH1*: TPS ≥ 50% 28.57%, 4/14; others: TPS ≥ 50% 11.61%, 41/353). (D) Within *NOTCH1* Sq cases also has high proportion of PD‐L1 expression (*NOTCH1*: TPS ≥ 50% 33.33%, 4/12; others: TPS ≥ 50% 25.00%, 16/24). Inside the fourteen *NOTCH1* co‐mutant cases, remarkedly, the *FGF/FGFR1/NOTCH1* within *RB1* variant group has a dramatically higher inner‐tumor CD8+ T cell infiltration [(E) left column, CD8: Orange, pan‐CK: Gray, scale bar = 50 μM; (F) **p < 0.01]. More inner‐tumor CD8+PD‐1‐LAG3‐ non‐exhausted T cells intensity is in within *RB1* variant group [E middle column, no green and magenta, PD‐1: Green, L AG3: Magenta, scale bar = 50 μM; (G) ****p < 0.001] and CD8+PD‐1+LAG3‐ exhausted T cells [E middle column, green and no magenta, PD‐1: Green, L AG3: Magenta, scale bar = 50 μM; (H) **p < 0.01]. Furthermore, the inner‐tumor CD69+CD103+ tissue‐resident memory T (T_RM_) cells, are at significant high frequencies in *FGF/FGFR1/NOTCH1* within *RB1* variant group. [E right column, yellow and red, CD69: Yellow, CD103: Red, scale bar = 50 μM; (I) *p < 0.05].

Ultimately, PV group HE slides are collected and analyzed by AI‐based model to reveal the microenvironment features. Interestingly, compared to the progressed cases, the non‐progressed ones have higher level of TLS formation, including both immature and mature ones (Figure 5A). While there is no big difference of TLS number between cases within *FGF* and *FGFR* variants (Figure 5B). It seems that the co‐mutant genes account for microenvironment remodeling in patients with good prognosis. According to statistics, co‐mutations in *FGF/FGFR* and *NOTCH1* (3.81%, 14/367, 8 PV and 6 VUS) are associated with significantly higher PD‐L1 expression ratio compared to cases without *NOTCH1* co‐mutations (with *NOTCH1* co‐mutations: Tumor proportion score (TPS) ≥ 50% 28.57%, 4/14; others: TPS ≥ 50% 11.61%, 41/353, Figure 5C). *NOTCH1* The Sq samples with *NOTCH1* co‐mutations have higher proportion of PD‐L1 expression (with *NOTCH1* co‐mutations TPS ≥ 50% 33.33%, 4/12; others: TPS ≥ 50% 25.00%, 16/24, Figure 5D). Inside the fourteen *NOTCH1* co‐mutant cases, we divided and compared between within *RB1* variant group (4/14) and without *RB1* variant group (10/14). Remarkedly, the *FGF/FGFR1/NOTCH1* within *RB1* variant group has a dramatically higher inner‐tumor CD8+ T cell infiltration (Figure 5E,F), following more CD8+PD‐1‐LAG3‐ non‐exhausted T cells (Figure 5E,G) and CD8+PD‐1+LAG3‐ exhausted T cells (Figure 5E,H). Furthermore, the CD69+CD103+ tissue‐resident memory T (T_RM_) cells, the important drivers of inflammation, are at significant high frequencies in *FGF/FGFR1/NOTCH1* within *RB1* variant group. (Figure 5E,I). Thus, it is obvious that the *FGF/FGFR1/NOTCH1* within *RB1* variant subtype tends to recruit an inflamed immune microenvironment, which might be a potential benefit population for anti‐PD‐1 therapy. This might support a pro‐immunotherapy role of *NOTCH1* gene in *FGF/FGFR* alteration cases.

## 4 Discussion

Recently, FGF ligands [11, 12, 13, 14, 15] and their receptors [13, 14, 15, 16, 17, 18] can serve as potential biomarkers in lung cancer. Additionally, treatment with FGFR inhibitors, including pemigatinib and erdafitinib can increases the survival rate of Non–small cell lung cancer (NSCLC) patients [18, 26]. The basic FGFR family has four active members including FGFR1, FGFR2, FGFR3, and FGFR4. Their activation plays a crucial role in cancer initiation [19, 20, 21, 22, 23, 24, 25]. However, the contributions of the *FGF/FGFR* signally pathway to patient survival, clinicopathological features, and characteristics of the tumor microenvironment remain poorly understood. Especially, the roles of *FGF* alterations have been scarcely investigated to date.

A total 4656 lung cancer cases examined by NGS are included in this study. Compared to the previous pan‐cancer study, [30, 31] we provide a comprehensive analysis about *FGF/FGFR* alteration in large‐scale Asian NSCLC cohort. About 10.27% (478/4656) patients harboring *FGF/FGFR* alteration are discussed on variant diversity in detail. We first find the *FGF/FGFR* variant is mainly in squamous cell carcinoma (Sq) rather than adenocarcinoma (Ad), which reveals a potential benefit of Sq from inhibitor treatment like pemigatinib and erdafitinib [18, 26]. Then, the obvious higher risk of smoking exposure, invasion behavior, and later pStage all promise an aggressive behavior in PV group. A significant shorter OS and DFS are shown in PV cases compared to VUS ones (Figure 2B,C). However, different variant subtypes might contribute to different outcome. Hence, we analyze the variant subtypes in depth. *FGFR* fusion seems to have better prognosis, while *FGF* amplification and *FGF*/*FGFR* combined alteration tumor suppressor gene co‐mutation (Figure 2A and Table S1, *p* < 0.0001).

Survival analysis reveal that *FGF* PV is an independent factor for poor prognosis, with a high hazard ratio for both OS and DFS. Conversely, *FGFR* variant is not a predictor for poor prognosis in PV group. The *FGF/FGFR* novel drug development is better to pay more attention on *FGF* variant. Besides, *FGFR3‐TACC3* fusion has a considerable ratio of the squamous carcinoma (83.33%, 5/6, Table S5), which may explain why the smoking ratio is in common.

Furthermore, despite the variant diversity, we also talk about the co‐mutation diversity in *FGF/FGFR* signaling alteration. The *FGF19/CCND1* co‐amplify is a smoking‐related event and leads to poor differentiation. This provides a latent drug target of neighboring genes on 11p13 region. Another similar pair is *FGF*/*FGFR* and *KRAS*. Co‐mutation gene *KRAS* reveals the high smoking exposure and poor prognosis risk in *FGF/FGFR* variant. *FGF*/*FGFR/KRAS* co‐mutation is a remarkable and independent prognosis factor in short DFS. Smokers might take advantage from integrating *FGF/FGFR* and *KRAS* target therapy.

Moreover, the non‐progressed cases display a high level of TLS formation, including both immature and mature ones. While there is no big difference of TLS number between cases within *FGF* and *FGFR* variant, which indicates the role of co‐mutation gene on microenvironment remodeling in patients with good prognosis. High PD‐L1 is frequent in *FGF/FGFR* and *NOTCH1* co‐mutant group, which implies the potential sensitivity to immunotherapy. To better uncover the potential effect of *NOTCH1* co‐mutation on immunotherapy, we also describe the surrounding microenvironment. The *FGF/FGFR1/NOTCH1* within *RB1* variant group has a dramatically inflamed microenvironment, showing higher inner‐tumor CD8+ T cell infiltration. Especially, the CD8+PD‐1+LAG3‐ exhausted T cells are predominant, which suggests a potential benefit from anti‐PD‐1 therapy [32, 33, 34, 35]. Furthermore, this group recruits the CD69+CD103+ tissue‐resident memory T (T_RM_) cells at a high frequency. These cells help form an inflamed microenvironment contributed to favorable clinical outcome [36, 37, 38]. According to the clinicopathological analysis, *NOTCH1* co‐mutation comes across with an astonishing high smoking exposure ratio (92.86%, 13/14) and high Sq ratio (85.71%, 12/14, Table S6). The smoking statues might promote inflamed microenvironment formation, which is more susceptible to immunotherapy [39, 40]. Simultaneously, the *RB1* gene itself would enhance sensitivity to immunotherapy by impacting immunological features and remodeling microenvironment [41].

To be honest, we still have some limitations. The survival analysis also reveals a favorable prognosis tendency in *NOTCH1* co‐mutant group rather than others (Figure S1A–D). But the follow‐up time of *NOTCH1* co‐mutant group is too short to drive a solid conclusion, because most cases just receive surgery in 2023. In addition, the *FGF/FGFR/NOTCH1* within *RB1* variant group shows a slightly high ratio of STAS, the mechanism remains unknown (Table S7). In addition, *FGFR* fusion also show a better outcome tendency compared to *FGFR* no fusion group, especially the *FGFR1* amplification group (Figure S1E,F). The follow‐up time for some cases is too short to draw a solid conclusion. These will be the future direction when we have longer observation time and enroll enough cases.

In sum, we summarized the mutation diversity of *FGF/FGFR* alteration in NSCLC, and further analysis of the survival and clinicopathological indicators in different variant subgroups. Besides, we analyzed the co‐mutation genes of *FGF/FGFR* variant to enrich the knowledge of *FGF/FGFR* signaling.

## 5 Conclusion

Given the above, our study provides a comprehensive analysis about *FGF/FGFR* alteration in a large‐scale NSCLC cohort of Asian patients with NSCLC, and it is observed that *FGF/FGFR* alterations are more prevalent in squamous cell carcinoma. Both *FGF* PV and *KRAS* mutations act as the independent risk factors of poor prognosis, and combined against these targets can significantly improve patient outcomes. To the best of our knowledge, this is the first report to describe the role of *NOTCH1/RB1* co‐mutations in the development of an inflammatory microenvironment, suggesting  the potential benefits of immunotherapy in these cases.

## Author Contributions

**Ziling Huang:** conceptualization, funding acquisition, formal analysis, investigation, methodology, and writing – original draft. **Leyao Li and Xu Cai:** data curation, formal analysis, investigation, methodology, and validation. **Shen Wang:** methodology. **Yun Jia:** methodology. **Yuan Li:** conceptualization, funding acquisition, project administration, supervision, and writing – review and editing.

## Ethics Statement

This retrospective study was approved by The Ethics Committee of the Fudan University Shanghai Cancer Center (No. 050432‐4‐2307E). Written informed consent was obtained from patients prior to study commencement.

## Conflicts of Interest

The authors declare no conflicts of interest.

## Supporting information


Data S1.

## Data Availability

All datasets included in the current study are available from the corresponding author on reasonable request.
