# Supplementary material for: A Comprehensive Analysis of FGF/FGFR Signaling Alteration in NSCLC: Implications in Prognosis and Microenvironment
Source: Thorac Cancer. 2025 Feb 25;16(4):e70016. doi: 10.1111/1759-7714.70016 (PMC11860276; doi:10.1111/1759-7714.70016)
Supplement: Supplementary file 1 — Data S1. [file TCA-16-e70016-s001.docx]

**Supplementary Figure** A. Longer OS is seen in *NOTCH1* co-mutation PV group compared to the without one. The survival rate is 100.00% in *NOTCH1* PV group while 77.65% in other PV group (*p*=0.2318). B. Longer DFS is seen in *NOTCH1* co-mutation PV group compared to the without one. The survival rate is 87.50% in *NOTCH1* PV group while 66.86% in other PV group (*p*=0.5676). C. Slightly longer OS is seen in *NOTCH1* co-mutation VUS group compared to the without one. The survival rate is 100.00% in *NOTCH1* VUS group while 95.17% in other VUS group (*p*=0.6959). D. Slightly longer DFS is seen in *NOTCH1* co-mutation VUS group compared to the without one. The survival rate is 100.00% in *NOTCH1* VUS group while 94.45% in other VUS group (*p*=0.6040). E. Longer OS is seen in *FGFR* fusion PV group compared to the no fusion one, especially the *FGFR1* amplification. The survival rate is 100.00% in *FGFR* fusion group and *FGFR2/3*^amp+mut^ group while 87.16% in *FGFR1* amplification group (*p*=0.2598). F. Longer DFS is seen in *FGFR* fusion group compared to the no fusion one, especially the *FGFR1* amplification. The survival rate is 100.00% in *FGFR* fusion group and *FGFR2/3*^amp+mut^ group while 81.98% in *FGFR1* amplification group (*p*=0.1653).


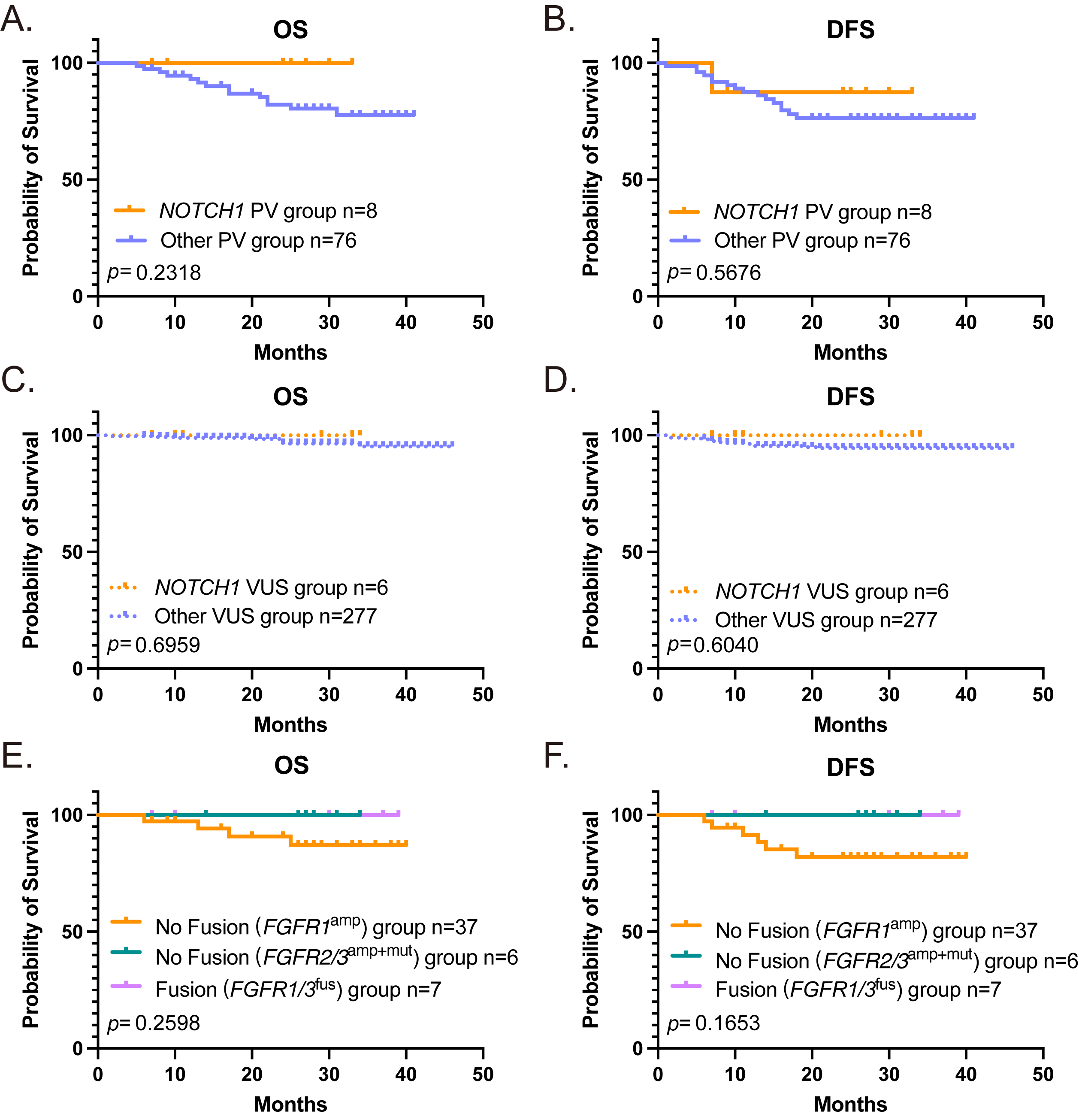


**Supplementary Table 1. The summary of *FGF/FGFR* variant subtypes in 367 surgically resected specimens.**

| **Characteristics** | **Total**  **(n=367)** | **≥2 FGFR**  **(n=1)** | **FGF Amplification**  **(n=30)** | **FGF SNV (n=52)** | **FGF+FGFR (n=7)** | **FGFR Amplification**  **(n=38)** | **FGFR Fusion**  **(n=9)** | **FGFR SNV**  **(n=230)** | ***p* value^a^** |
| --- | --- | --- | --- | --- | --- | --- | --- | --- | --- |
| **Group** |  |  |  |  |  |  |  |  | <0.001 |
| PV group | 84 (22.89%) | 1 (0.00%) | 30 (100.00%) | 0 (0.00%) | 4 (57.14%) | 38 (100.00%) | 7 (77.78%) | 4 (1.74%) |  |
| VUS group | 283 (77.11%) | 0 (0.00%) | 0 (0.00%) | 52 (100.00%) | 3 (42.86%) | 0 (0.00%) | 2 (22.22%) | 226 (98.26%) |  |
| **Histology** |  |  |  |  |  |  |  |  | /^b^ |
| Adenocarcinoma | 263 (71.66%) | 0 (0.00%) | 11 (36.67%) | 44 (84.62%) | 3 (42.86%) | 7 (18.42%) | 3 (33.3%) | 195 (84.78%) |  |
| Squamous carcinoma | 76 (20.71%) | 1 (100.00%) | 16 (53.33%) | 4 (7.69%) | 3 (42.86%) | 26 (68.42%) | 5 (55.56%) | 21 (9.13%) |  |
| Other | 28 (7.63%) | 0 (0.00%) | 3 (10.00%) | 4 (7.69%) | 1 (14.29%) | 5 (13.16%) | 1 (11.1%) | 14 (6.09%) |  |
| **Driven Mutations** |  |  |  |  |  |  |  |  | /^b^ |
| Within ≥2 Driven Mutations | 16 (4.36%) | 0 (0.00%) | 2 (6.67%) | 2 (3.85%) | 2 (28.57%) | 1 (2.63%) | 0 (0.00%) | 9 (3.91%) |  |
| Within 1 Driven Mutations | 233 (63.49%) | 0 (0.00%) | 16 (53.33%) | 30 (57.69%) | 3 (42.86%) | 13 (34.21%) | 5 (55.56%) | 166 (72.17%) |  |
| Without Driven Mutations | 118 (32.15%) | 1 (100.00%) | 12 (40.00%) | 20 (38.46%) | 2 (28.57%) | 24 (63.16%) | 4 (44.44%) | 55 (23.91%) |  |
| **Tumor Suppressor Mutations** |  |  |  |  |  |  |  |  | /^b^ |
| Within ≥2 Tumor Suppressor Mutations | 35 (9.54%) | 0 (0.00%) | 2 (6.67%) | 8 (15.38%) | 0 (0.00%) | 4 (10.53%) | 0 (0.00%) | 21 (9.13%) |  |
| Within 1 Tumor Suppressor Mutations | 165 (44.96%) | 1 (100.00%) | 25 (83.33%) | 19 (36.54%) | 5 (71.43%) | 29 (76.32%) | 6 (66.67%) | 80 (34.78%) |  |
| Without Tumor Suppressor Mutations | 167 (45.50%) | 0 (0.00%) | 3 (10.00%) | 25 (48.08%) | 2 (28.57%) | 5 (13.16%) | 3 (33.33%) | 129 (56.09%) |  |
| ***KRAS*** |  |  |  |  |  |  |  |  | 0.133 |
| *FGF/FGFR/KRAS* Co-alteration Group | 36 (9.81%) | 0 (0.00%) | 6 (20.00%) | 8 (15.38%) | 1 (14.29%) | 1 (2.63%) | 0 (0.00%) | 20 (8.70%) |  |
| Others | 331 (90.19%) | 1 (100.00%) | 24 (80.00%) | 44 (84.62%) | 6 (85.71%) | 37 (97.37%) | 9 (100.00%) | 210 (91.30%) |  |
| **PD-L1 TPS** |  |  |  |  |  |  |  |  | /^b^ |
| TPS<1% | 242 (65.94%) | 1 (100.00%) | 20 (66.67%) | 32 (61.54%) | 4 (57.14%) | 20 (52.63%) | 3 (33.33%) | 162 (70.43%) |  |
| 1%≤TPS<50% | 80 (21.80%) | 0 (0.00%) | 5 (16.67%) | 10 (19.23%) | 2 (28.57%) | 10 (26.32%) | 4 (44.44%) | 49 (21.30%) |  |
| TPS≥50% | 45 (12.26%) | 0 (0.00%) | 5 (16.67%) | 10 (19.23%) | 1 (14.29%) | 8 (21.05%) | 2 (22.22%) | 19 (8.26%) |  |
| **Smoking history** |  |  |  |  |  |  |  |  | <0.001 |
| Absent | 182 (49.59%) | 0 (0.00%) | 7 (23.33%) | 23 (44.23%) | 3 (42.86%) | 8 (21.05%) | 4 (44.44%) | 137 (59.57%) |  |
| Present | 185 (50.41%) | 1 (100.00%) | 23 (76.67%) | 29 (55.77%) | 4 (57.14%) | 30 (78.95%) | 5 (55.56%) | 93 (40.43%) |  |
| **Progression** |  |  |  |  |  |  |  |  | <0.001 |
| Progress Group | 33 (8.99%) | 0 (0.00%) | 10 (33.33%) | 4 (7.69%) | 2 (28.57%) | 6 (15.79%) | 1 (11.11%) | 10 (4.35%) |  |
| Non-Progress Group | 334 (91.01%) | 1 (100.00%) | 20 (66.67%) | 48 (92.31%) | 5 (71.43%) | 32 (84.21%) | 8 (88.89%) | 220 (95.65%) |  |
| **Death** |  |  |  |  |  |  |  |  | <0.001 |
| Death Group | 24 (6.54%) | 0 (0.00%) | 7 (23.33%) | 3 (5.77%) | 3 (42.86%) | 4 (10.5%) | 1 (11.11%) | 6 (2.61%) |  |
| Alive Group | 343 (93.46%) | 1 (100.00%) | 23 (76.67%) | 49 (94.23%) | 4 (57.14%) | 34 (89.47%) | 8 (88.89%) | 224 (97.39%) |  |

Data are presented as n (%).

Abbreviations: *FGFR*, fibroblast growth factor receptor; *FGF*, fibroblast growth factor; SNV, single nucleotide variation; PV, pathogenic variant; VUS, variant of only uncertain significance; *KRAS*, Kirsten rat sarcoma viral oncogene homolog; PD-L1, programmed cell death protein-1 ligand TPS, tumor proportion scoring.

^a^: Fisher's exact probability test is used for calculation.

^b^: Cannot be computed because there is insufficient memory.

**Supplementary Table 2. The clinicopathological characteristics in 84 surgically resected specimens within *FGF/FGFR* PV cases.**

| **Characteristics** | **Total (n=84)** | ***FGF* (n=34)** | ***FGFR* (n=50)** | ***p* value** |
| --- | --- | --- | --- | --- |
| **Age** |  |  |  | 1.000^a^ |
| Median (IQR) | 64.0 (58.0-69.0) |  |  |  |
| ≤64.0 | 46 (54.76%) | 19 (55.88%) | 27 (54.00%) |  |
| >64.0 | 38 (45.24%) | 15 (44.12%) | 23 (46.00%) |  |
| **Sex** |  |  |  | 0.761^a^ |
| Female | 13 (15.48%) | 6 (17.65%) | 7 (14.00%) |  |
| Male | 71 (84.52%) | 28 (82.35%) | 43 (86.00%) |  |
| **Tumor size** |  |  |  | 0.979^a^ |
| <2.5 (25th percentile) | 16 (19.05%) | 6 (17.65%) | 10 (20.00%) |  |
| ≥2.5 and <3.5 (50th percentile) | 18 (21.43%) | 8 (23.53%) | 10 (20.00%) |  |
| ≥3.5 and <5.0  (75th percentile) | 28 (33.33%) | 11 (32.35%) | 17 (34.00%) |  |
| ≥5.0 | 22 (26.19%) | 9 (26.47%) | 13 (26.00%) |  |
| **Histology** |  |  |  | 0.328^a^ |
| Other | 12 (14.29%) | 4 (11.76%) | 8 (16.00%) |  |
| Adenocarcinoma | 20 (25.00%) | 11 (32.35%) | 9 (18.00%) |  |
| Squamous carcinoma | 52 (60.71%) | 19 (55.88%) | 33 (66.00%) |  |
| **Differentiation** |  |  |  | 0.635^c^ |
| Well differentiated | 2 (2.38%) | 0 (0.00%) | 2 (4.00%) |  |
| Moderately differentiated | 17 (20.24%) | 8 (23.53%) | 9 (18.00%) |  |
| Poorly differentiated | 65 (77.38%) | 26 (76.47%) | 39 (78.00%) |  |
| **pStage** |  |  |  | 0.547^c^ |
| Ⅰ | 44 (52.38%) | 16 (47.06%) | 28 (56.00%) |  |
| Ⅱ | 13 (15.48%) | 5 (14.71%) | 8 (16.00%) |  |
| Ⅲ | 23 (27.38%) | 10 (29.41%) | 13 (26.00%) |  |
| Ⅳ | 4 (4.76%) | 3 (8.82%) | 1 (2.00%) |  |
| **TNM_T stage** |  |  |  | 0.745^c^ |
| T1 | 30 (35.71%) | 12 (35.29%) | 18 (36.00%) |  |
| T2 | 34 (40.48%) | 14 (41.18%) | 20 (40.00%) |  |
| T3 | 13 (15.48%) | 4 (11.76%) | 9 (18.00%) |  |
| T4 | 7 (8.33%) | 4 (11.76%) | 3 (6.00%) |  |
| **TNM_N stage** |  |  |  | 0.269^c^ |
| N0 | 63 (71.43%) | 27 (79.41%) | 36 (72.00%) |  |
| N1 | 8 (9.52%) | 1 (2.94%) | 7 (14.00%) |  |
| N2 | 13 (15.48%) | 6 (17.65%) | 7 (14.00%) |  |
| **Venous/lymphatic invasion** |  |  |  | 0.654^a^ |
| Absent | 52 (61.90%) | 20 (58.82%) | 32 (64.00%) |  |
| Present | 32 (38.10%) | 14 (41.18%) | 18 (36.00%) |  |
| **Nerve invasion** |  |  |  | 1.000^b^ |
| Absent | 74 (88.10%) | 30 (88.24%) | 44 (88.00%) |  |
| Present | 10 (11.90%) | 4 (11.76%) | 6 (12.00%) |  |
| **Pleural invasion** |  |  |  | 0.761^a^ |
| Absent | 71 (84.52%) | 28 (82.35%) | 43 (86.00%) |  |
| Present | 13 (15.48%) | 6 (17.65%) | 7 (14.00%) |  |
| **STAS** |  |  |  | 0.530^b^ |
| Absent | 73 (86.90%) | 31 (91.18%) | 42 (84.00%) |  |
| Present | 11 (13.10%) | 3 (8.82%) | 8 (16.00%) |  |
| **Smoking history** |  |  |  | 1.000^a^ |
| Absent | 18 (21.43%) | 7 (20.59%) | 11 (22.00%) |  |
| Present | 66 (78.57%) | 27 (79.41%) | 39 (78.00%) |  |
| **PD-L1 TPS** |  |  |  | 0.466^a^ |
| TPS>1% | 48 (57.14%) | 22 (64.71%) | 26 (52.00%) |  |
| 1%≤TPS<50% | 20 (23.81%) | 6 (17.65%) | 14 (28.00%) |  |
| TPS≥50% | 16 (19.05%) | 6 (17.65%) | 10 (20.00%) |  |

Data are presented as n (%).

Abbreviations: PV, pathogenic variant; VUS, variant of only uncertain significance; IQR, interquartile range; TNM, tumor-node-metastasis; STAS, spread through air spaces; PD-L1, programmed cell death protein-1 ligand TPS, tumor proportion scoring.

^a^: Pearson Chi-Square is used for calculation.

^b^: Continuity correction is used for calculation.

^c^: Fisher's exact probability test is used for calculation.

**Supplementary Table 3. Univariate analysis of clinicopathological features for overall survival (OS) in *FGF/FGFR* PV cases.**

| **Characteristics** | **Numbers of cases** | **HR (95% CI)** | ***p* value** |
| --- | --- | --- | --- |
| **Variant subtype1** |  |  | 0.026 |
| *FGFR* PV | 50 | 1.000 |  |
| *FGF* PV | 34 | 3.747 (1.173~11.970) |  |
| **Variant subtype2** |  |  | 0.209 |
| Other PV | 76 | 1.000 |  |
| *KRAS* PV | 8 | 2.274 (0.631~8.199) |  |
| **Variant subtype3** |  |  | 0.439 |
| Other PV | 76 | 1.000 |  |
| *NOTCH1* PV | 8 | 0.043 (0.000~123.954) |  |
| **Age** |  |  | 0.090 |
| Median (IQR) | 64.0 (58.0-69.0) |  |  |
| ≤64.0 | 46 | 1.000 |  |
| >64.0 | 38 | 2.584 (0.863~7.732) |  |
| **Sex** |  |  | 0.861 |
| Female | 13 | 1.000 |  |
| Male | 71 | 1.144 (0.256~5.115) |  |
| **Tumor size** |  |  | 0.867 |
| <2.5 (25th percentile) | 16 | 1.000 |  |
| ≥2.5 and <3.5 (50th percentile) | 18 | 0.563 (0.094~3.368) |  |
| ≥3.5 and <5.0 (75th percentile) | 28 | 1.112 (0.278~4.450) |  |
| ≥5.0 | 22 | 0.867 (0.175~4.299) |  |
| **Histology** |  |  | 0.007 |
| Other | 12 | 1.000 |  |
| Adenocarcinoma | 20 | 0.151 (0.030~0.751) |  |
| Squamous carcinoma | 52 | 0.190 (0.061~0.594) |  |
| **Differentiation** |  |  | 0.892 |
| Well differentiated | 3 | 1.000 |  |
| Moderately differentiated | 16 | 10568.713 (0.000~3.734E+123) |  |
| Poorly differentiated | 65 | 7986.000 (0.000~2.818E+123) |  |
| **pStage** |  |  | 0.027 |
| Ⅰ | 44 | 1.000 |  |
| Ⅱ | 13 | 0.662 (0.077~5.668) |  |
| Ⅲ | 23 | 2.283 (0.660~7.895) |  |
| Ⅳ | 4 | 7.832 (1.860~32.978) |  |
| **TNM_T stage** |  |  | 0.975 |
| T1 | 30 | 1.000 |  |
| T2 | 34 | 1.296 (0.411~4.090) |  |
| T3 | 13 | 1.297 (0.251~6.716) |  |
| T4 | 7 | 0.000 (0.000~0.000) |  |
| **TNM_N stage** |  |  | 0.061 |
| N0 | 63 | 1.000 |  |
| N1 | 8 | 0.000 (0.000~0.000) |  |
| N2 | 13 | 3.768 (1.254~11.321) |  |
| **Venous/lymphatic invasion** |  |  | 0.060 |
| Absent | 52 | 1.000 |  |
| Present | 32 | 2.774 (0.958~8.032) |  |
| **Nerve invasion** |  |  | 0.375 |
| Absent | 74 | 1.000 |  |
| Present | 10 | 0.041 (0.000~48.055) |  |
| **Pleural invasion** |  |  | 0.416 |
| Absent | 71 | 1.000 |  |
| Present | 13 | 0.430 (0.056~3.291) |  |
| **STAS** |  |  | 0.525 |
| Absent | 73 | 1.000 |  |
| Present | 11 | 0.517 (0.067~3.957) |  |
| **Smoking history** |  |  | 0.571 |
| Absent | 18 | 1.000 |  |
| Present | 66 | 0.715 (0.224~2.281) |  |
| **PD-L1 TPS** |  |  | 0.758 |
| TPS<1% | 39 | 1.000 |  |
| 1%≤TPS<50% | 19 | 0.759 (0.205~2.808) |  |
| TPS≥50% | 16 | 0.582 (0.126~2.694) |  |

Abbreviations: HR, hazard ratio; CI, confidence interval; *FGFR*, fibroblast growth factor receptor; *FGF*, fibroblast growth factor; *KRAS*, Kirsten rat sarcoma viral oncogene homolog; PV, pathogenic variant; IQR, interquartile range; TNM, tumor-node-metastasis; STAS, spread through air spaces; PD-L1, programmed cell death protein-1 ligand TPS, tumor proportion scoring.

**Supplementary Table 4. Univariate analysis of clinicopathological features for disease-free survival (DFS) in *FGF/FGFR* PV and *KRAS* PV cases.**

| **Characteristics** | **Numbers of cases** | **HR (95% CI)** | ***p* value** |
| --- | --- | --- | --- |
| **Variant subtype1** |  |  | 0.014 |
| *FGFR* PV | 50 | 1.000 |  |
| *FGF* PV | 34 | 3.431 (1.287~9.147) |  |
| **Variant subtype2** |  |  | 0.037 |
| Other PV | 76 | 1.000 |  |
| *KRAS* PV | 8 | 3.279 (1.076~9.991) |  |
| **Variant subtype3** |  |  | 0.536 |
| Other PV | 76 | 1.000 |  |
| *NOTCH1* PV | 8 | 0.529 (0.070~3.978) |  |
| **Age** |  |  | 0.953 |
| Median (IQR) | 64.0 (58.0-69.0) |  |  |
| ≤64.0 | 46 | 1.000 |  |
| >64.0 | 38 | 0.972 (0.383~2.465) |  |
| **Sex** |  |  | 0.655 |
| Female | 13 | 1.000 |  |
| Male | 71 | 1.398 (0.321~6.083) |  |
| **Tumor size** |  |  | 0.540 |
| <2.5 (25th percentile) | 16 | 1.000 |  |
| ≥2.5 and <3.5 (50th percentile) | 18 | 0.305 (0.059~1.575) |  |
| ≥3.5 and <5.0 (75th percentile) | 28 | 0.587 (0.179~1.927) |  |
| ≥5.0 | 22 | 0.714 (0.207~2.465) |  |
| **Histology** |  |  | 0.034 |
| Other | 12 | 1.000 |  |
| Adenocarcinoma | 20 | 0.338 (0.095~1.199) |  |
| Squamous carcinoma | 52 | 0.251 (0.087~0.725) |  |
| **Differentiation** |  |  | 0.965 |
| Well differentiated | 2 | 1.000 |  |
| Moderately differentiated | 17 | 9558.898 (0.000~2.704E+110) |  |
| Poorly differentiated | 65 | 8262.271 (0.000~2.334E+110) |  |
| **pStage** |  |  | 0.002 |
| Ⅰ | 44 | 1.000 |  |
| Ⅱ | 13 | 0.635 (0.074~5.440) |  |
| Ⅲ | 23 | 3.912 (1.309~11.694) |  |
| Ⅳ | 4 | 12.269 (2.902~51.868) |  |
| **TNM_T stage** |  |  | 0.943 |
| T1 | 30 | 1.000 |  |
| T2 | 34 | 0.963 (0.349~2.657) |  |
| T3 | 13 | 0.731 (0.152~3.520) |  |
| T4 | 7 | 0.578(0.071~4.699) |  |
| **TNM_N stage** |  |  | 0.002 |
| N0 | 63 | 1.000 |  |
| N1 | 8 | 0.787 (0.100~6.214) |  |
| N2 | 13 | 5.397 (2.067~14.094) |  |
| **Venous/lymphatic invasion** |  |  | 0.021 |
| Absent | 52 | 1.000 |  |
| Present | 32 | 3.071 (1.189~7.933) |  |
| **Nerve invasion** |  |  | 0.382 |
| Absent | 74 | 1.000 |  |
| Present | 10 | 0.406 (0.054~3.054) |  |
| **Pleural invasion** |  |  | 0.302 |
| Absent | 71 | 1.000 |  |
| Present | 13 | 0.345 (0.046~2.596) |  |
| **STAS** |  |  | 0.637 |
| Absent | 73 | 1.000 |  |
| Present | 11 | 1.348 (0.390~4.658) |  |
| **Smoking history** |  |  | 0.833 |
| Absent | 18 | 1.000 |  |
| Present | 66 | 0.888 (0.292~2.697) |  |
| **PD-L1 TPS** |  |  | 0.594 |
| TPS<1% | 39 | 1.000 |  |
| 1%≤TPS<50% | 19 | 0.779 (0.251~2.419) |  |
| TPS≥50% | 16 | 0.469 (0.105~2.098) |  |

Abbreviations: HR, hazard ratio; CI, confidence interval; *FGFR*, fibroblast growth factor receptor; *FGF*, fibroblast growth factor; *KRAS*, Kirsten rat sarcoma viral oncogene homolog; PV, pathogenic variant; IQR, interquartile range; TNM, tumor-node-metastasis; STAS, spread through air spaces; PD-L1, programmed cell death protein-1 ligand TPS, tumor proportion scoring.

**Supplementary Table 5. The clinicopathological characteristics in *FGFR3-TACC3* fusion cases.**

| **Characteristics** | **Total (n=14)** | ***FGFR3-TACC3* fusion (n=6)** | **Other *FGFR* fusion (n=8)** | ***p* value*^a^*** |
| --- | --- | --- | --- | --- |
| **Age** |  |  |  |  |
| Median (IQR) | 64.5 (56.0-70.5) |  |  | 1.000 |
| ≤64.5 | 7 (50.00%) | 3 (50.00%) | 4 (50.00%) |  |
| >64.5 | 7 (50.00%) | 3 (50.00%) | 4 (50.00%) |  |
| **Sex** |  |  |  | 0.209 |
| Female | 3 (21.43%) | 0 (0.00%) | 3 (37.50%) |  |
| Male | 11 (78.57%) | 6 (100.00%) | 5 (62.50%) |  |
| **Tumor size** |  |  |  | 0.113 |
| <2.2  (25th percentile) | 3 (21.43%) | 0 (0.00%) | 3 (37.50%) |  |
| 2.2 and <3.9  (50th percentile) | 4 (28.57%) | 1 (16.67%) | 3 (37.50%) |  |
| ≥3.9 and <5.6  (75th percentile) | 4 (28.57%) | 2 (33.33%) | 2 (25.50%) |  |
| ≥5.6 | 3 (21.43%) | 3 (50.00%) | 0 (0.00%) |  |
| **Histology** |  |  |  | 0.065 |
| Other | 3 (21.43%) | 1 (16.67%) | 2 (25.0%) |  |
| Adenocarcinoma | 4 (28.57%) | 0 (0.00%) | 4 (50.00%) |  |
| Squamous carcinoma | 7 (50.00%) | 5 (83.33%) | 2 (25.00%) |  |
| **Differentiation** |  |  |  | 0.209 |
| Well differentiated | 0 (0.00%) | 0 (0.00%) | 0 (0.00%) |  |
| Moderately differentiated | 3 (21.43%) | 0 (0.00%) | 3 (37.50%) |  |
| Poorly differentiated | 11 (78.57%) | 6 (100.00%) | 5 (62.50%) |  |
| **pStage** |  |  |  | 0.334 |
| Ⅰ | 5 (35.71%) | 1 (16.67%) | 4 (50.00%) |  |
| Ⅱ | 4 (28.57%) | 3 (50.00%) | 1 (12.50%) |  |
| Ⅲ | 5 (35.71%) | 2 (33.33%) | 3 (37.50%) |  |
| **Ⅳ** | 0 (0.00%) | 0 (0.00%) | 0 (0.00%) |  |
| **TNM_T stage** |  |  |  | 0.020 |
| T1 | 6 (42.86%) | 0 (0.00%) | 6 (75.00%) |  |
| T2 | 4 (28.57%) | 3 (50.00%) | 1 (12.50%) |  |
| T3 | 4 (28.57%) | 3 (50.00%) | 1 (12.50%) |  |
| T4 | 0 (0.00%) | 0 (0.00%) | 0 (0.00%) |  |
| **TNM_N stage** |  |  |  | 0.450 |
| N0 | 8 (57.14%) | 3 (50.00%) | 5 (62.50%) |  |
| N1 | 2 (14.29%) | 2 (33.33%) | 0 (0.00%) |  |
| N2 | 4 (28.57%) | 1 (16.67%) | 3 (37.50%) |  |
| **Venous/lymphatic invasion** |  |  |  | 1.000 |
| Absent | 8 (57.14%) | 3 (50.00%) | 5 (62.50%) |  |
| Present | 6 (42.86%) | 3 (50.00%) | 3 (37.50%) |  |
| **Nerve invasion** |  |  |  | 0.429 |
| Absent | 13 (92.86%) | 5 (83.33%) | 8 (100.00%) |  |
| Present | 1 (7.14%) | 1 (16.67%) | 0 (0.00%) |  |
| **Pleural invasion** |  |  |  | 0.429 |
| Absent | 13 (92.86%) | 5 (83.33%) | 8 (100.00%) |  |
| Present | 1 (7.14%) | 1 (16.67%) | 0 (0.00%) |  |
| **STAS** |  |  |  | /^b^ |
| Absent | 14 (100.0%) | 6 (100.0%) | 8 (100.0%) |  |
| Present | 0 (0.00%) | 0 (0.00%) | 0 (0.00%) |  |
| **Smoking history** |  |  |  | 0.031 |
| Absent | 5 (35.71%) | 0 (0.00%) | 5 (62.50%) |  |
| Present | 9 (64.29%) | 6 (100.00%) | 3 (37.50%) |  |
| **PD-L1 TPS** |  |  |  | 0.800 |
| TPS<1% | 6 (42.86%) | 2 (33.33%) | 4 (50.00%) |  |
| 1%≤TPS<50% | 5 (35.71%) | 3 (50.00%) | 2 (25.00%) |  |
| TPS≥50% | 3 (21.43%) | 1 (16.67%) | 2 (25.00%) |  |

Data are presented as n (%).

Abbreviations: *FGFR3*, fibroblast growth factor receptor 3; *TACC3*, transforming acidic acid coiled-coil protein 3; IQR, interquartile range; TNM, tumor-node-metastasis; STAS, spread through air spaces; PD-L1, programmed cell death protein-1 ligand TPS, tumor proportion scoring.

^a^: Fisher's exact probability test is used for calculation.

^b^: It is not possible to compute the statistic because STAS is a constant.

**Supplementary Table 6. The clinicopathological characteristics in *FGF/FGFR* and *Notch1* co-mutation cases.**

|  | **Total (n=367)** | ***Notch1* (n=14)** | **Other (n=353)** | ***p* value** |
| --- | --- | --- | --- | --- |
| **Age** |  |  |  | 0.587^a^ |
| Median (IQR) | 63 (55.0-68.0) |  |  |  |
| ≤63 | 194 (52.86%) | 6 (42.8%) | 188 (53.25%) |  |
| >63 | 173 (47.14%) | 8 (57.14%) | 165 (46.74%) |  |
| **Sex** |  |  |  | 0.001^a^ |
| Female | 146 (39.78%) | 0 (0.00%) | 146 (41.36%) |  |
| Male | 221 (60.22%) | 14 (100.00%) | 207 (58.64%) |  |
| **Tumor size** |  |  |  | 0.002^c^ |
| <1.3  (25th percentile) | 83 (22.62%) | 0 (0.00%) | 83 (23.51%) |  |
| ≥1.3 and <2.0  (50th percentile) | 79 (21.53%) | 0 (0.00%) | 79 (22.38%) |  |
| ≥2.0 and <3.5  (75th percentile) | 106 (28.88) | 6 (42.86%) | 100 (28.33%) |  |
| ≥3.5 | 99 (26.98%) | 8 (57.14%) | 91 (25.78%) |  |
| **Histology** |  |  |  | 0.000^b^ |
| Adenocarcinoma | 263 (71.66%) | 1 (7.14%) | 262 (74.22%) |  |
| Squamous carcinoma | 76 (20.71%) | 12 (85.71%) | 64 (18.13%) |  |
| Other | 28 (7.63%) | 1 (7.14%) | 27 (7.65%) |  |
| **Differentiation** |  |  |  | 0.152^c^ |
| Well differentiated | 54 (14.71%) | 1 (7.14%) | 53 (15.01%) |  |
| Moderately differentiated | 124 (14.29%) | 2 (20.24%) | 122 (34.56%) |  |
| Poorly differentiated | 189 (78.57%) | 11 (77.38%) | 178 (50.42%) |  |
| **pStage** |  |  |  | 0.689^c^ |
| Ⅰ | 265 (72.21%) | 9 (64.28%) | 256 (72.52%) |  |
| Ⅱ | 36 (9.81%) | 2 (14.29%) | 34 (9.63%) |  |
| Ⅲ | 55 (14.99%) | 23 (21.43%) | 52 (14.73%) |  |
| Ⅳ | 11 (3.00%) | 0 (0.00%) | 11 (3.12%) |  |
| **TNM_T stage** |  |  |  | 0.020^c^ |
| T1 | 252 (68.66%) | 5 (35.71%) | 247 (69.97%) |  |
| T2 | 80 (21.80%) | 7 (50.00%) | 73 (20.68%) |  |
| T3 | 22 (5.99%) | 2 (14.29%) | 20 (5.67%) |  |
| T4 | 13 (3.54%) | 0 (0.00%) | 13 (3.68%) |  |
| **TNM_N stage** |  |  |  | 0.312^c^ |
| N0 | 304 (82.83%) | 11 (78.57%) | 293 (83.00%) |  |
| N1 | 23 (6.27%) | 0 (0.00%) | 23 (6.52%) |  |
| N2 | 40 (10.90%) | 3 (21.43%) | 37 (10.48%) |  |
| **Venous/lymphatic invasion** |  |  |  | 0.621^b^ |
| Absent | 270 (73.57%) | 9 (64.29%) | 261 (73.94%) |  |
| Present | 97 (26.43%) | 5 (35.71%) | 92 (26.06%) |  |
| **Nerve invasion** |  |  |  | 0.039^c^ |
| Absent | 346 (94.28%) | 11 (78.57%) | 335 (94.90%) |  |
| Present | 21 (5.72%) | 3 (21.43%) | 18 (5.10%) |  |
| **Pleural invasion** |  |  |  | 0.936^b^ |
| Absent | 330 (89.92%) | 12 (85.71%) | 318 (90.08%) |  |
| Present | 37 (10.08%) | 2 (14.29%) | 35 (9.92%) |  |
| **STAS** |  |  |  | 0.848^b^ |
| Absent | 283 (77.11%) | 10 (71.43%) | 273 (77.34%) |  |
| Present | 84 (22.89%) | 4 (28.57%) | 80 (22.66%) |  |
| **Smoking history** |  |  |  | 0.002^a^ |
| Absent | 182 (49.59%) | 1 (7.14%) | 181 (51.27%) |  |
| Present | 185 (50.41%) | 13 (92.86) | 172 (48.73%) |  |
| **PD-L1 TPS** |  |  |  | 0.154^c^ |
| TPS<1% | 242 (65.94%) | 7 (50.00%) | 235 (66.57%) |  |
| 1%≤TPS<50% | 80 (21.80%) | 3 (21.43%) | 77 (21.81%) |  |
| TPS≥50% | 45 (12.26%) | 4 (28.57%) | 41 (11.61%) |  |

Data are presented as n (%).

Abbreviations: IQR, interquartile range; TNM, tumor-node-metastasis; STAS, spread through air spaces; PD-L1, programmed cell death protein-1 ligand TPS, tumor proportion scoring.

^a^: Pearson Chi-Square is used for calculation.

^b^: Continuity correction is used for calculation.

^c^: Fisher's exact probability test is used for calculation.

**Supplementary Table 7. The clinicopathological characteristics in *FGF/FGFR/NOTCH1* within *RB1* variant cases*.***

|  | **Total (n=14)** | ***FGF/FGFR/NOTCH1***  **^with^ *^RB1^* variant (n=4)** | ***FGF/FGFR/NOTCH1***  **^without^ *^RB1^* ^variant^ (n=10)** | ***p* value*^a^*** |
| --- | --- | --- | --- | --- |
| **Age** |  |  |  | 0.559 |
| **Median (IQR)** | 64.50 (59.3-71.3) |  |  |  |
| ≤64.5 | 7 (50.00%) | 1 (25.00%) | 6 (60.00%) |  |
| >64.5 | 7 (50.00%) | 3 (75.00%) | 4 (40.00%) |  |
| **Sex** |  |  |  | /^b^ |
| Female | 0 (0.00%) | 0 (0.00%) | 0 (0.00%) |  |
| Male | 14 (100.00%) | 4 (100.00%) | 10 (100.00%) |  |
| **Tumor size** |  |  |  | 0.595 |
| <2.5  (25th percentile) | 0 (0.00%) | 0 (0.00%) | 0 (0.00%) |  |
| ≥2.5 and <3.5  (50th percentile) | 6 (42.86%) | 2 (50.00%) | 4 (40.00%) |  |
| ≥3.5 and <4.6  (75th percentile) | 5 (35.71%) | 2 (50.00%) | 3 (30.00%) |  |
| ≥4.6 | 3 (21.43%) | 0 (0.00%) | 3 (30.00%) |  |
| **Histology** |  |  |  | 0.066 |
| Adenocarcinoma | 1 (7.14%) | 1 (25.00%) | 0 (0.00%) |  |
| Squamous carcinoma | 12 (85.71%) | 2 (50.00%) | 10 (100.00%) |  |
| Other | 1 (7.14%) | 1 (25.00%) | 0 (0.00%) |  |
| **Differentiation** |  |  |  | 1.000 |
| Well differentiated | 1 (7.14%) | 0 (0.00%) | 1 (10.00%) |  |
| Moderately differentiated | 2 (14.29%) | 0 (0.00%) | 2 (20.00%) |  |
| Poorly differentiated | 11 (78.57) | 4 (100.00%) | 7 (70.00%) |  |
| **pStage** |  |  |  | 1.000 |
| Ⅰ | 9 (64.29%) | 3 (75.00%) | 6 (60.00%) |  |
| Ⅱ | 2 (14.29%) | 0 (0.00%) | 2 (20.00%) |  |
| Ⅲ | 3 (21.43%) | 1 (25.00%) | 2 (20.00%) |  |
| Ⅳ | 0 (0.00%) | 0 (0.00%) | 0 (0.00%) |  |
| **TNM_T stage** |  |  |  | 0.580 |
| T1 | 5 (35.71%) | 1 (25.00%) | 4 (40.00%) |  |
| T2 | 7 (50.00%) | 3 (75.00%) | 4 (40.00%) |  |
| T3 | 2 (14.29 | 0 (0.00%) | 2 (20.00%) |  |
| T4 | 0 (0.00%) | 0 (0.00%) | 0 (0.00%) |  |
| **TNM_N stage** |  |  |  | 1.000 |
| N0 | 11 (78.57) | 3 (75.00%) | 8 (80.00%) |  |
| N1 | 0 (0.00%) | 0 (0.00%) | 0 (0.00%) |  |
| N2 | 3 (21.43%) | 1 (25.00%) | 2 (20.00%) |  |
| **Venous/lymphatic invasion** |  |  |  | 0.580 |
| Absent | 9 (64.29%) | 2 (50.00%) | 7 (70.00%) |  |
| Present | 5 (35.71%) | 2 (50.00%) | 3 (30.00%) |  |
| **Nerve invasion** |  |  |  | 0.505 |
| Absent | 11 (78.57%) | 4 (100.00%) | 7 (70.00%) |  |
| Present | 3 (21.43%) | 0 (0.00%) | 3 (30.00%) |  |
| **Pleural invasion** |  |  |  | 0.505 |
| Absent | 12 (85.71%) | 3 (75.00%) | 9 (90.00%) |  |
| Present | 4 (14.29%) | 1 (25.00%) | 1 (10.00%) |  |
| **STAS** |  |  |  | 0.041 |
| Absent | 10 (71.43%) | 1 (25.00%) | 9 (90.00%) |  |
| Present | 4 (28.57%) | 3 (75.00%) | 1 (10.00%) |  |
| **Smoking history** |  |  |  | 1.000 |
| Absent | 1 (7.14%) | 0 (0.00%) | 1 (10.00%) |  |
| Present | 13 (92.86% ) | 4 (100.00%) | 9 (90.00%) |  |
| **PD-L1 TPS** |  |  |  | 0.357 |
| TPS<1% | 7 (50.00%) | 3 (75.00%) | 4 (40.00%) |  |
| 1%≤TPS<50% | 3 (21.43%) | 1 (25.00%) | 2 (20.00%) |  |
| TPS≥50% | 4 (28.57) | 0 (0.00%) | 4 (40.00%) |  |

Data are presented as n (%).

Abbreviations: *FGF*, fibroblast growth factor; *FGFR*, fibroblast growth factor receptor; *RB1*, Retinoblastoma 1; IQR, interquartile range; TNM, tumor-node-metastasis; STAS, spread through air spaces; PD-L1, programmed cell death protein-1 ligand TPS, tumor proportion scoring.

^a^: Fisher's exact probability test is used for calculation.

^b^: It is not possible to compute the statistic because STAS is a constant.
